# Industrial Assessment Tools of Risks Associated with Wastewater and Water Technologies

**DOI:** 10.1021/acsestwater.5c00839

**Published:** 2025-09-16

**Authors:** Anna Trubetskaya, Aine Hennessy, Ian Ryan, Ken Stockil, Colm Gaskin

**Affiliations:** † Department of Engineering, University of Limerick, Castletroy, V94T9PX Limerick, Ireland; ‡ Department of Biosciences, Nord University, 8026 Bodø, Norway; § 20FIFTYpartners, Innovation House, Lonsdale Rd, National Technology Park, Castletroy, V94W8K8 Limerick, Ireland

**Keywords:** industrial risk assessment tools, wastewater and water technologies, risk frameworks, water resource management

## Abstract

This work explores the current landscape of industrial risk assessment tools for wastewater and water technologies, emphasizing the need for integrated, multirisk frameworks that address environmental, technological, regulatory, and socioeconomic dimensions. Leveraging on over 7,000 literature sources and two European (EU) case studies in the chemical and paper manufacturing sectors, this study identifies key gaps in existing risk methodologies and highlights the importance of aligning risk assessments with evolving EU directives and climate goals. Integration of two detailed case studies in the Irish food industry and at a Portuguese pulp and paper manufacturing site provides practical validation of the proposed frameworks. The case studies categorized risk assessments at micro- and macrolevels guiding the establishment of future frameworks. In the long term perspective, the inclusion of digital technologies aims to enhance predictive capabilities and resilience of the water and wastewater sectors. The findings advocate for an inclusive and adaptive risk management approach that bridges operational detail with strategic oversight, ensuring sustainable and compliant water resource management within industrial settings.

## Graphical abstract

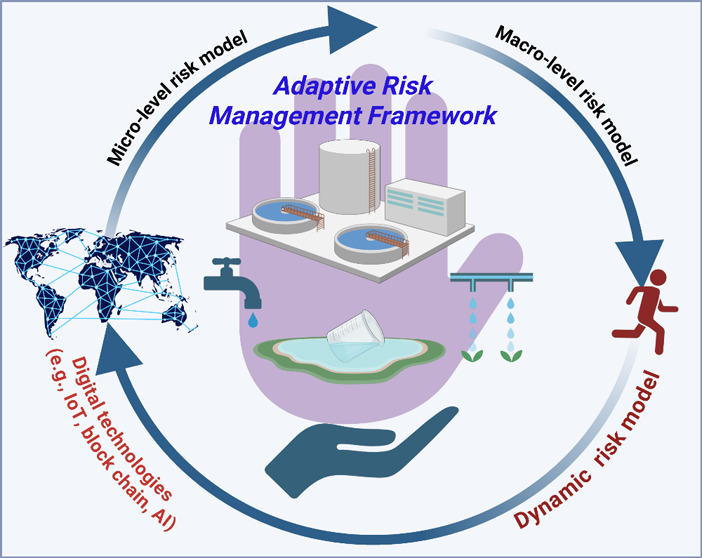

## 1 Introduction

In the water and wastewater sectors, risk assessments are increasingly recognized as essential for ensuring safe, sustainable, and compliant operations. The adoption of new technologies, evolving regulatory frameworks, and emerging environmental threats such as Per- and Polyfluoroalkyl Substances (PFAS), pharmaceuticals, and climate-induced stressors necessitate structured evaluations of potential risks.^1,2^ Key drivers influencing technology adoption include cost savings, reputational benefits, technological advancement, and resource scarcity. While regulatory compliance and sustainability goals are acknowledged, they are often deprioritized due to inconsistent enforcement.^3^ Additionally, supply chain disruptions, maintenance demands, and cultural readiness significantly shape risk perceptions and decisions around implementing new technologies.^4^

A significant expansion of risk assessment methodologies has been observed over the last seven years, particularly related to systemic risks that affect business operations, supply chains, regulatory landscapes, and long-term strategic planning. Despite significant progress in risk assessment frameworks, new climate challenges have emerged, including increasing emissions, the unpredictability of ecosystems under climate change, and the decreasing availability of resources due to geopolitical and environmental shifts. Another challenge associated with industrial risk assessment is the absence of a comprehensive multi-risk framework evaluation that accounts for pollutant exposure, natural capital, finances, novel technologies, and regulations.

Existing literature identifies climate risk assessment as a tool to inform management decisions regarding individual production facilities. In addition, it is recognized as a tool to assess and mitigate against the likelihood of environmental contamination issues.^5^ According to the European Water Framework Directive (WFD), the risks associated with the identification of priority hazardous substances outweigh other risks.^6^ The WFD and its supporting directives, such as the Groundwater Directive (GWD) and the Environmental Quality Standards Directive (EQSD), establish a comprehensive framework for protecting water quality across Europe. Within this framework, the concept of water quality risk has emerged as a critical dimension of environmental risk assessment. Water quality risk refers to the potential for contamination or degradation of water resources, particularly those linked to industrial operations. This includes risks deriving from seasonal variability in river flows, declining groundwater levels, reduced snowpack and glacier melt, and increased frequency of droughts, all of which are being exacerbated by climate change. For example, in southern Europe, prolonged droughts have led to significant reductions in river discharge and reservoir storage, directly impacting industrial operations that depend on consistent water supply.^7^ As such, water quality risk is no longer viewed in isolation but is increasingly integrated into broader environmental and climate risk assessments, reinforcing the need for proactive and holistic management strategies.

The risks associated with industrial water encompass various phases of contaminant migration, transformation, and accumulation within the hydrosphere, e.g., surface water bodies, groundwater, and their eventual discharge into the sea and ocean, unified global water system, etc.^2^ Thus, risk frameworks for industrial water risk assessments must also consider source vulnerability. This includes evaluating catchment-scale water balances, aquifer recharge rates, and competition with other water users (e.g., agriculture, municipalities). In water-stressed regions, overabstraction from aquifers has led to critical drawdowns, threatening long-term water security for industrial zones. Recent updates to the EU Water Directives have included new pollutants to address emerging environmental threats, such as PFAS, pharmaceuticals, and pesticides. PFAS contaminants for a group of man-made chemicals that are persistent in the environment and have been linked to adverse health effects, necessitating an update of the WFD due to their extremely toxic impacts on animals and humans.^8^ Certain pharmaceuticals, such as antibiotics and hormones, have been added due to their potential impact on aquatic ecosystems and human health.^9^ Additional pesticide active substances included to enhance the protection of water quality from agricultural runoff.^10^ According to the water directives and recent updates, priority substances and hazardous pollutants pose significant risks to both surface water and groundwater, particularly from environmental, human health, and socioeconomic perspectives.^11,12^ Industrial water risk assessments are crucial in this context, as they facilitate the identification of potential pollution sources, the evaluation of risks associated with industrial activities, and the development of strategies to manage and mitigate these risks to both surface water and groundwater utilized by industrial sites. The alignment with the EU’s water protection policies ensures that industries comply with regulations and contribute to the sustainable management of water resources. Some EU countries–such as Germany, Denmark, Sweden, Netherlands, etc.–proactively support projects to implement industrial water risk analysis more than others, due to stricter national regulations and stronger governmental support for the integration of new water technologies and education.^13^ Spain and Italy, facing chronic water scarcity have implemented industrial water reuse programs and seasonal abstraction limits to manage supply risks. In contrast, northern European countries focus more on flood risk and stormwater management, which are also part of the broader water quantity risk spectrum. While there is significant research in energy technology risk assessments, water and wastewater-related risks remain critical, but may not always be as immediately visible or urgent to the public.^14^ Previous research has shown that energy issues often receive more attention than water-related challenges, due to their direct impact on economic activities and daily life.^15^ Historically, the focus in water management has been on ensuring supply and quality rather than risk assessment. This has started to change with the introduction of frameworks, such as Water Safety Plans (WSPs), but the shift is still ongoing in EU policies. While the direct cost of water is often perceived by consumers as lower than that of energy, public and academic attention to water-related risks is diminished.^16^ Another challenge in discussing risk assessment for water and wastewater systems is the scarcity of standardized methodologies.^17^

Previous reviews and research studies have often focused on specific aspects such as operational risks in wastewater treatment plants, risks related to public health, environmental impacts, and infrastructure resilience.^18,19^ However, water-related risks encompass not only these areas but also strategic socioeconomic, legislative, and human resource factors. The Environmental Quality Standard Directive 2008/105/EC sets limits for concentrations of certain pollutants in surface waters while it also addresses legislative and socioeconomic factors by ensuring that water quality standards are met, which can impact public health and economic activities.^20^ Effective water management, in compliance with regulatory standards, involves the assessment of the risk significance that is often linked to the risk materiality model.

The risk materiality model can be used to identify and prioritize a broad range of significant risks to water quality and quantity including environmental risks (e.g., pollution from industrial activities or extreme weather events like flooding), legislative risks (e.g., noncompliance with EU directives), and socioeconomic risks (e.g., impacts on public health and economic activities). Recognizing water quantity risk as a critical challenge for industrial operations—driven by growing global demand, aging infrastructure, and the intensifying impacts of climate change—such models enable industries to assess both water scarcity and excess water risks.^21^ Water, being not only a vital resource but also a source of significant business risk, requires industries to adopt novel management strategies to ensure resilience and sustainability.^22^ For example, it can highlight the need for alternative water sources, storage infrastructure, or seasonal production adjustments. By integrating water quantity into risk assessments, industries can better align with EU directives and ensure long-term operational resilience.^23^ In addition, resources can be allocated more effectively to address the most critical risks identified under the EU Water Directives, ensuring that efforts are focused on areas with the highest potential impact on water quality, water quantity (including both scarcity and excess), and regulatory compliance. It can also support strategic planning by highlighting key areas where intervention is needed. For example, if the materiality assessment identifies PFAS contamination as a high-risk factor, targeted measures can be implemented to monitor and mitigate this pollutant in accordance with the EQSD.^24^

This article focuses on the review of recent literature sources aiming to synthesize the current advancements and challenges in quantitative and qualitative risks associated with industrial water. Different risk assessment frameworks and links between them will be discussed, including the broader implications of the risk assessments from environmental, technological, strategic, legislative, and socioeconomic perspectives. The definition of risks will be explored in this article, incorporating multiperspective considerations from EU policymakers, who view water-related risks as potential threats to the achievement of political, strategic, and operational objectives. Two case studies will be performed using food processing, paper and pulp manufacturing sites across Europe. In addition, by examining the latest regulatory updates and technological innovations, this article provides a comprehensive overview of how industrial water risk assessments can contribute to sustainable water management and compliance with EU standards. This article addresses the following scientific questions:

- **RQ1:** How can industrial water and wastewater risks be comprehensively assessed using multidimensional frameworks?
- **RQ2:** What are the most effective tools and strategies for mitigating risks in industrial water and wastewater systems?

This article presents a comprehensive synthesis of multidimensional risk assessment frameworks for industrial water and wastewater systems, uniquely supported by case studies that illustrate the integration of micro- and macrolevel perspectives, advanced technologies, and regulatory insights in alignment with EU sustainability goals.

## 2 Method

This article employs a systematic approach to evaluate industrial assessment tools for risks associated with wastewater and water technologies. Data was collected from Scopus and Web of Science academic databases, industry reports, and regulatory/policy documents, focusing on publications from 2019 to 2024 totaling 7430 literature sources, as shown in Figure 1.

**Figure 1:**
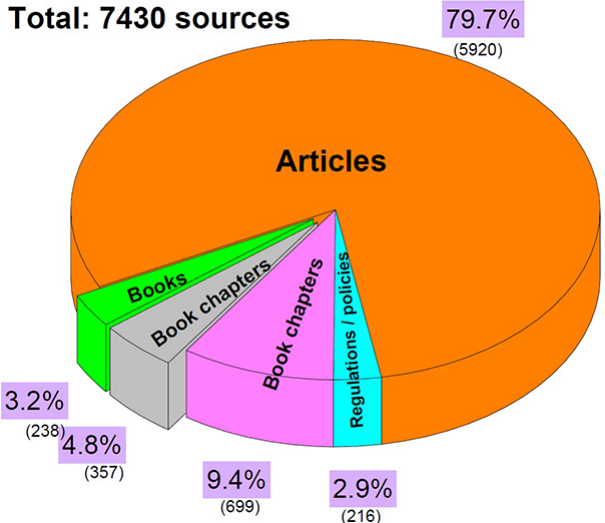
Wastewater and water technology risk assessment literature sources identified in Scopus and Web of Science databases.

The database search identified the field of industrial risk assessment and water technologies as interdisciplinary perspectives from environmental science, engineering, policy, and management holistic solutions. The regulatory documents and policies showed the smallest number of available literature sources on water and wastewater risk assessments. Articles represented the highest available literature category consisting of 70–80% research articles. One of the main gaps in the literature on wastewater and water technology risk assessment is the need for comprehensive multirisk assessment frameworks that integrate various types of risks from the research and methodology perspectives.

Literature research in Figure 1 was guided by the keywords such as “risk assessment frameworks”, “water & wastewater technology”, “water-related regulations”, “industrial risk categories”, and “risk management tools”. The evaluation of these keywords was performed against various risk categories – operational, technical, strategic, environmental, human resource capital, financial, reputational, and regulatory risks as shown in Figure 2.

**Figure 2:**
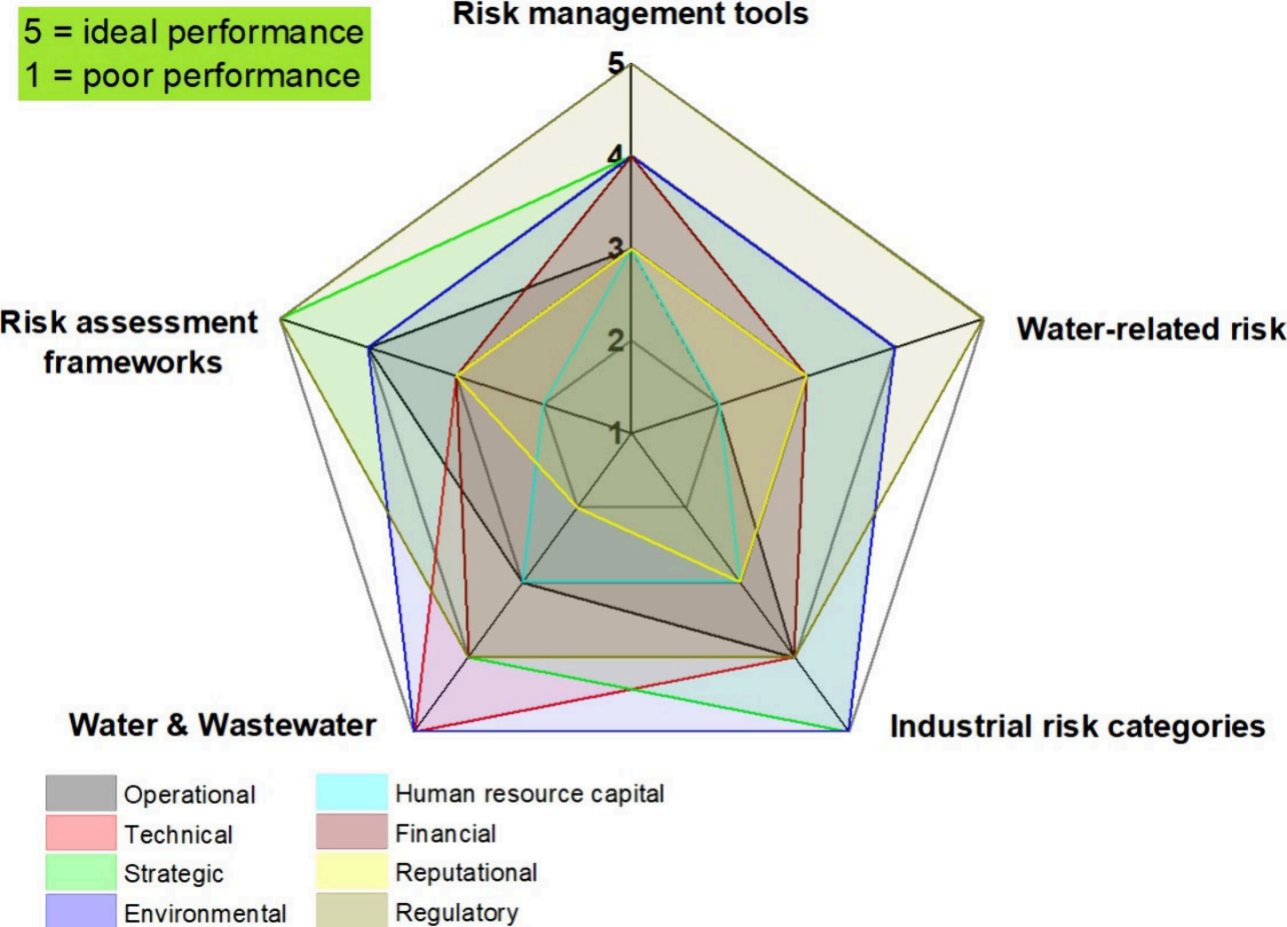
Methodology of selected literature sources according to the risk categories and keyword analysis.

A total of 50 sources for each keyword were selected to develop criteria for the evaluation of its effectiveness. Literature sources were selected based on relevance, methodological rigor, and contribution to understanding industrial water risk assessments. Data extraction involved capturing key information about each assessment tool, including its purpose, methodology, and outcomes. The quality of the studies was assessed using PRISMA guidelines, ensuring a thorough and unbiased synthesis. Qualitative analysis identified recurring themes and patterns, while quantitative methods, including meta-analysis, quantified the effectiveness of different tools. Each keyword was scored on a scale of 1 to 5 based on its relevance and effectiveness in addressing these risk categories. Then, the performance of each keyword across the different risk categories was compared by using a scoring system identifying patterns and trends in its effectiveness. This semiquantitative scoring system, illustrated in Figure 2, assigned values from 1 to 5 to each keyword based on its relevance and effectiveness across eight industrial risk categories. The radar chart visualizes these scores, enabling both qualitative pattern recognition and quantitative comparison of keyword performance across operational, technical, strategic, environmental, human resource, financial, reputational, and regulatory dimensions.

Two case studies of food processing, pulp and paper manufacturing sites in Europe provided practical insights into the application of these tools. The data analysis from both industrial sites was conducted by using internal communications and detailed reports. Information was gathered through comprehensive surveys and in-depth interviews, all structured within the data management plan of the Horizon EU project “RESURGENCE” (Grant Agreement Number of 101138097). The surveys were designed to capture a wide range of perspectives from various stakeholders, while the interviews provided deeper insights into specific operational challenges and risk factors. By integrating these methods, the project was able to compile a robust data set that facilitated a thorough assessment of industrial risks associated with wastewater and water technologies.

## 3 Results and Discussion

Before delving into a detailed analysis, this section presents the key findings and interpretations derived from the literature review and case studies. It aims to contextualize the subsequent discussion by first exploring how risk is defined and understood by EU policymakers in the water and wastewater sectors.

### 3.1 Risk Definitions from the Perspective of EU Policymakers

No single definition of the term “risk” can be deemed universally correct, as it must align with the diverse values of different those who define it.^25^ However, the precise definition of the multifaceted term “risk” is essential for the water and wastewater technology sector addressing its unique challenges and complexities.^26^

In the context of European policies, particularly the Corporate Sustainability Reporting Directive (CSRD), it is important to distinguish between the concepts of risk and impact as defined under the double materiality approach.^27^ Here, impacts refer to situations in which organizations may cause effects on the surrounding environment and adjacent communities (an inside-out perspective), while risks denote situations in which the external environment, especially climate-related factors, may affect the organization (an outside-in perspective). It is common, though conceptually imprecise within this framework to see examples such as noncompliance with emission limit values (ELVs), or negative industrial events described as risks. According to the CSRD, these should instead be categorized as impacts, reflecting the organization’s influence on its environment rather than vulnerabilities to external factors.

First, ensuring public health and safety is paramount. Contaminants in drinking water, such as pathogens, chemicals and heavy metals, and PFAS can cause serious health issues.^28^ Effective risk definitions help identify and mitigate these threats, ensuring that industrial water treatment processes are robust and reliable. Second, pollution from untreated or inadequately treated wastewater can harm aquatic ecosystems and biodiversity. This is particularly important in the context of climate change, which increases the frequency and severity of extreme weather events, leading to flooding and infrastructure damage.^1^ Third, the water and wastewater sectors rely on advanced technologies for treatment, monitoring, and distribution. Defining risks accurately helps in identifying vulnerabilities in these technologies, such as system failures or cyber-attacks ensuring the resilience and security of these technologies for continuous service delivery.^29^ Additionally, the sector is subject to stringent regulations aimed at protecting public health and the environment.^30^ Noncompliance with these regulations can result in legal penalties, financial losses, and reputational damage to various industrial sectors.^31^

The accurate risk definitions allow for better prioritization and allocation of resources.^4^ By understanding the most significant risks, utilities can focus their efforts and investments on areas that will have the greatest impact on safety and reliability to maintain the efficient and cost-effective operations in the water and wastewater sector.^32^

In addition, all risks in the water and wastewater sector are often interdependent.^33^ As an example, environmental risks can exacerbate public health risks, and technological failures can lead to regulatory noncompliance. A clear and comprehensive definition of risk helps to understand and manage these complex relationships, providing a structured approach to effective risk management.

Initially, the definition of “risk” in the water and wastewater sector was broad and varied, reflecting the diverse values and priorities of different stakeholders.^34^ This lack of a unified definition made it challenging to develop consistent risk management strategies. In the early 2000s, the introduction of frameworks like the Water Safety Plans (WSPs) by the World Health Organization (WHO) marked a significant change emphasizing the importance of identifying and managing risks to ensure safe water consumption.^35^ They provided a structured risk management approach focusing on prevention measures and continuous drinking water quality monitoring.^36^ The risk in the wastewater sector was mainly classified as biological and chemical categories.^37^ In recent years, the definition of risk has further evolved to incorporate the concept of resilience involving integrated water management and risk-based approaches. The risks were classified based on the type of stressors affecting the wastewater systems.^38^ Chronic stressors are well-known, recurrent, and often predictable, e.g., urbanization and population growth, aging infrastructure, climate variability and change, equipment failures, and changes in water use and reuse patterns. In contrast, acute stressors are unpredictable and uncommon and can have severe consequences, e.g., floods, earthquakes, disease outbreaks, terrorist attacks, and extreme weather. Such classification of stressors led to the formulation of technological, regulatory, environmental, reputational, financial, and socioeconomic risks.^39^ This classification also includes the ability of water and wastewater systems to withstand and recover from various cyberattacks.^40^ In the last five years, the increasing recognition of interdependencies between different types of risks has led to a more holistic view of risk management that includes governance, sustainability, and compliance. This comprehensive and collaborative approach considers the interconnected nature of risks and the need for coordinated efforts across different sectors and stakeholders.^41^ The compliance risks ensure that water management practices adhere to local, national, and international regulations and standards, while the interests and rights of all stakeholders, particularly those from marginalized communities, are equally considered for inclusive decision-making processes.^42^ This led to the prominence of the strategic risk category as strategic risk management considers the interdependence between diverse sectors, e.g., food, water, energy.^43^ Strategic risk management considers the interdependence between different sectors requiring coordinated efforts to address complex challenges.^44^ Up to this day, the risk management in the water and wastewater sector is characterized by structured frameworks, resilience planning, and a holistic understanding of interdependencies with the focus on proactive and continuous improvement, and collaboration among various stakeholders to ensure the operational safety and reliability.^45,46^ The extension of strategic risk categories connected the risk management approach to Lean Six Sigma (LSS) methodologies, which focused on improving efficiency, reducing waste, and enhancing quality. The LSS approach became highly relevant to the water and wastewater sector for optimizing resource use, reducing costs, and improving overall system performance.^47^

### 3.2 Risk Assessment at Micro- and Macrolevels

Risk associated with industrial water involves evaluating potential hazards and implementing strategies to mitigate these risks. This process can be approached at both micro- and macrolevels, each with distinct definitions, risk mitigation strategies, and frameworks.

At the **microlevel**, risk assessment focuses on specific components and processes within both water intake, treatment activities, and effluent emissions. This involves identifying potential hazards associated with individual equipment, chemical usage, and operational procedures.^48^ The primary goal is to comply with regulations. In the case of water withdrawal, it is important to ensure that the standards and authorized volumes of water are met; in the case of effluent treatment, it is important to ensure compliance with the ELVs. In addition, the objectives include continuous improvement of processes, ensuring maximum possible efficiency, always acting within a context of safety for operations, the environment, and nature. Risk mitigation strategies at this level often include regular maintenance, real-time monitoring, and immediate corrective actions to address any detected anomalies using the Plan, Do, Act, Check (PDCA) framework as suggested in ISO14001.^49^ For instance, the use of hazard identification and risk assessment methods helps in pinpointing specific risks and managing them effectively using the Waste Water Treatment Plant (WWTP) case study in Ireland with LSS tools.^45^ Another example of a **microlevel** WWTP assessment is a dynamic failure risk analysis of a wastewater treatment and reclamation plant at the Moorchekhort Industrial Complex in Iran. This study utilized a Dynamic Bayesian Network (DBN) to capture complex interactions between failure factors and predict future failure likelihoods.^50^ Over a 15-year period, the DBN model identified crucial risk factors and proposed mitigation measures that significantly reduced the failure risk from 33% to 9%.

Risk management at the **macrolevel** includes a comprehensive analysis of the interactions of industrial processes with the environment and communities. That is, it includes an assessment of physical and transitional climate risks related to water from an outside-in perspective as well as an assessment of the environmental impacts of water intake, treatment activities, and effluent emissions from an inside-out perspective. Risk assessment frameworks at the macrolevel include assessment of climate scenarios over different time horizons, assessment of the regulatory context, assessment of potential impacts from different perspectives - economic, environmental, social, reputational, etc.^51^ Mitigation strategies here might involve long-term planning, community engagement, and policy development to ensure sustainable operations and minimize adverse impacts. The macrolevel risk frameworks often require interdisciplinary approaches and collaboration among different sectors to effectively manage risks and will be discussed later in this work. Previous case studies indicated that only by combining the insights from both risk assessment levels, organizations can develop more effective and sustainable risk mitigation strategies.^52,53^

The macrolevel risk assessment analysis combined the economic, operational, social, and environmental impacts of water reuse in industrial parks in Germany.^54^ The findings revealed that the economic profitability of water reuse depends on wastewater effluent discharge and possible alternatives. Larger volumes of treated water reduced costs per cubic meter by up to 33%. The study also highlighted the importance of mitigating future water shortages to prevent potential socioeconomic problems for industry and society. Operationally, effective planning and evaluation of water reuse concepts were emphasized as crucial for sustainability and also enhancing the reputation of industrial parks by demonstrating environmental responsibility. Additionally, the study emphasized the need for robust IT and cybersecurity systems to manage and monitor water reuse processes at the industrial park effectively. This macrolevel risk assessment can be referred to the combination of Life Cycle Analysis (LCA) and Cost-Benefit Analysis (CBA) while the social and reputational sites of the water reuse system are quantitatively assessed using the likelihood and impact matrix.

### 3.3 Risk Mitigation Strategies

Various approaches and strategies were developed to address industrial water and wastewater risk-related challenges. This holistic perspective is essential for understanding how to effectively and dynamically safeguard water and wastewater resources and ensure their sustainability for future use on industrial sites. By exploration of advanced treatment technologies, nature-based solutions, policy and regulatory measures, and risk assessment tools, an overview of the current state of the risk mitigation strategies will be provided. Through a detailed examination of these risk mitigation strategies, definitions, the advantages, potential drawbacks, and the interconnectedness of different risk mitigation approaches are shown in Table 1.

**Table 1:** Risk Mitigation Strategies Including Their Definition, Advantages, Disadvantages, and Interconnectedness

Risk mitigation strategy	Definitions	Advantages	Disadvantages	Interconnectedness
Microlevel Risk Models				
IT and Cybersecurity Measures^55^	Implementing robust IT and cybersecurity practices is crucial to protect against cyber threats. This includes regular updates, network segmentation, and strong access controls to prevent unauthorized access	Protects against cyber threats ensure data integrity and confidentiality.	It can be costly and require continuous updates and monitoring.	Essential for all other strategies to ensure data security and operational integrity.
Advanced Treatment Technologies^56^	Utilizing advanced treatment processes such as ultrafiltration, UV disinfection, and advanced oxidation processes to ensure high-quality water treatment and reduce risks associated with contaminants.	Ensures high-quality water treatment reduces risks from contaminants.	High initial costs and maintenance requirements.	Supports sustainability practices and quality monitoring.
Regular Maintenance and Infrastructure Upgrades^57^	Ensuring that water and wastewater treatment facilities are regularly maintained and upgraded to handle increasing demand and emerging contaminants.	Improves system reliability and efficiency, reduces downtime.	It can be expensive and time-consuming.	Critical for emergency response plans and business continuity.
Redundancy and Backup Systems^58^	Having redundant systems and backup power supplies can help maintain operations during equipment failures or power outages. This ensures continuous service and minimizes downtime.	Ensure continuous operation, minimizes downtime.	Additional costs for implementation and maintenance.	Supports business continuity and emergency response plans.
Macrolevel Risk Models				
Quality Monitoring and Source Water Protection^59^	Continuous monitoring of water quality parameters helps detect and address contamination issues promptly. Advanced sensors and real-time data analytics can enhance monitoring capabilities.	Early detection of defects, improved water quality.	Requires investment in technology and training.	Integral to advanced treatment technologies and sustainability practices.
Sustainability Practices^60^	Implementation of environmentally friendly and resource-efficient methods to ensure long-term operational resilience, reduce environmental impact, and enhance the organization’s ability to adapt to changing conditions.	Reduces environmental impact, enhances resilience.	May require changes in operations and initial investment.	Supports all other strategies by promoting long-term sustainability.
Emergency Response Plans (EPR)^61^	Developing and regularly updating emergency response plans to address potential incidents such as chemical spills, natural disasters, or system failures.	Preparedness for emergencies reduces impact of incidents.	Requires regular updates and training.	Essential for business continuity and operational safety.
Business Continuity Response Plans (BCP)^62^	ensuring that an organization can maintain essential operations and quickly recover from disruptions by proactively planning, allocating resources, and implementing effective response and recovery procedures.	Maintains essential operations, quick recovery from disruptions.	Requires comprehensive planning and resources.	Interconnected with redundancy systems and emergency response plans.
Regulatory Compliance^63^	Staying up to date with local, national, and international regulations and standards to ensure compliance and avoid legal and financial penalties.	Avoids legal and financial penalties, ensures safe operations.	It can be complex and time-consuming to stay updated.	Supports all other strategies by ensuring compliance and safety.
Risk Assessment and Management^3^	Conducting regular risk assessments to identify potential hazards and implementing risk management strategies to mitigate identified risks.	Identifies and mitigates risks, improves safety and reliability.	Requires continuous monitoring and updating.	Integral to all other strategies for identifying and managing risks.
Training and Awareness Programs^64,65^	Engaging with the community and stakeholders to raise awareness about industrial water conservation, pollution prevention, and the importance of maintaining water quality.	Increasing awareness and engagement promotes best practices.	Requires ongoing effort and resources.	Supports all other strategies by promoting awareness and best practices.

The risk mitigation strategies can be categorized according to their macro- or microlevel of application. Macrolevel strategies ensure system-wide resilience and compliance with regulations, while microlevel strategies focus on the reliability and efficiency of specific components and processes. The comprehensive risk assessment framework addresses both immediate and long-term risk mitigation strategies to achieve resilience to future challenges.

In the context of **macrolevel** risk assessment, the risk mitigation strategies shown in Table 1 address large-scale risks that can impact the entire industrial water and wastewater system. Regulatory compliance ensures adherence to local, national, and international regulations to avoid legal and financial penalties, which are crucial for maintaining safe operations across the entire system. Implementing sustainable practices, such as water recycling and reuse, reduces the environmental footprint and conserves resources, aligning with global sustainability goals and enhancing the long-term viability of water systems. Emergency response plans are developed and regularly updated to address potential large scale incidents, such as chemical spills, natural disasters, or system failures, ensuring preparedness for significant disruptions.

At the **microlevel**, risk assessment focuses on specific components and processes within the industrial water and wastewater system. IT and cybersecurity measures protect individual control systems and data from cyber threats, involving regular updates, network segmentation, and strong access controls, to prevent unauthorized access. Advanced treatment technologies utilize specific treatment processes such as ultrafiltration and UV disinfection to ensure high-quality water treatment and reduce risks from contaminants at a granular level. Regular maintenance and infrastructure upgrades ensure that specific facilities and equipment are regularly maintained and upgraded to handle increasing demand and emerging contaminants, improving the reliability and efficiency at the component level. Redundancy and backup systems maintain operations during equipment failures or power outages, minimizing downtime for specific processes. Quality monitoring and source water protection involve the continuous tracking of water quality parameters to promptly detect and address contamination issues, using advanced sensors and real-time data analytics for specific monitoring tasks. Training and awareness programs engage stakeholders to raise awareness about industrial water conservation, pollution prevention, and maintaining water quality, promoting best practices at individual and team levels.

Understanding the advantages and limitations of each approach is crucial. Based on the summary in Table 1, a holistic risk management framework can be developed that incorporates the identified risk mitigation strategies. This framework should leverage the interconnectedness of the risk mitigation strategies to enhance the overall effectiveness of the risk assessment. For example, integrating IT and cybersecurity measures with quality monitoring systems can provide robust protection against cyber threats while ensuring high-quality water treatment.

Emphasizing the importance of continuous improvement in the framework is vital. Regularly updating the risk mitigation strategies through the systematic review process ensures that the risk management framework remains relevant and effective over time.^18^ Integrating Lean Six Sigma methodologies into this framework can further enhance its effectiveness and continuous improvement through systematically identifying inefficiencies, streamlining processes, and implementing corrective actions to mitigate risks.^66^ This LSS approach not only enhances the overall performance of the water and wastewater management system but also ensures that the framework remains agile and capable of adapting to new challenges.^67^ Engaging with the community, regulatory bodies, and industry experts can provide valuable feedback and support for implementing risk mitigation strategies in real life. This engagement can be facilitated through regular consultations, public forums, and collaborative workshops, ensuring that diverse perspectives are considered and that the framework benefits from a broad base of knowledge and experience. By fostering an environment of continuous learning and adaptation, the risk mitigation management framework can remain robust and responsive to the dynamic nature of industrial water and wastewater management and bring additional interconnectedness between various mitigation strategies.

### 3.4 Main Risk Assessment Frameworks

In the realm of water and wastewater technology, risk assessment frameworks play a crucial role in ensuring the safety and reliability of water systems. These frameworks help to identify, evaluate, and mitigate risks associated with water quality, infrastructure, and operational processes. The review work of ref 68 summarized the main risk management framework with respect to each category definition, principles, theories, and methods behind with the knowledge stand up to 2022. It discusses the application of qualitative, semiqualitative, and quantitative methods, including deterministic and probabilistic approaches, to identify potential hazards, calculate the probability of accidents, and assess the severity of consequences. The review also underscores the need for continuous development of risk assessment principles, theories, and base methods applicable to water supply systems.

In this article, Table 2 shows the main categories of risk assessment frameworks including their benefits, limitations, and previously used combinations with other frameworks. The Technology Risk Assessment framework, as discussed by refs 69 and 70, focuses on identifying and evaluating risks associated with technological components and innovations in water and wastewater systems assessing technological failures, cybersecurity threats, and the impact of new technologies on existing infrastructure. Complementing this, the IT Risk Framework, highlighted by refs 71 and 72, addresses risks such as data breaches, system downtimes, and software vulnerabilities, ensuring that IT infrastructure supports the overall safety and functionality of water systems.

**Table 2:** Summary of All Risk Assessment Frameworks in the Water and Wastewater Technology Sector

Model	Benefits	Limitations	Previous used in combination with other frameworks
Microlevel Risk Models			
Technology Risk Assessment^69,70^	Risks such as cybersecurity, system failure, data breaches identified, compliance and regulatory adherence, operational efficiency that standardizes the process, enhanced decision-making with the clear strategy, improved collaboration and communication, long-term planning and sustainability	Complex and Human Resources intense, dynamic technology change, quantification challenges of risks which lack the precision, siloed implementation across the organization, over-reliance on frameworks with sometimes emerging threats not covered by the framework	In combination with other models, for example, Control Objectives for Information and Related Technologies (COBIT), Factor Analysis of Information Risk (FAIR), International Organization for Standardization/International Electrotechnical Commission 27001 (ISO/IEC 27001), National Institute of Standards and Technology Risk Management Framework (NIST RMF), and Operationally Critical Threat, Asset, and Vulnerability Evaluation (OCTAVE), these frameworks have been used in wastewater and water assessment. Although Threat Assessment and Remediation Analysis (TARA) has been referenced, Technology Risk Assessment (TRA) has never been specifically applied to water-related risk assessment. The NIST Risk Management Framework (NIST RMF) has been predominantly used in the wastewater sector.
IT Risk Framework^71,72^	Common language for communication between business, IT, risk, and audit management, end-to-end guidance, complete risk profile enabling better resource utilization and risk prioritization, alignment with business goals, improved risk awareness, potential to be combined with other frameworks	Complexity, dynamic IT environment, quantification challenges, over-reliance on framework, siloed implementation	Combined with COBIT, NIST, ISO31000, FMEA, Cyber-Physical Systems (CPS), Environmental Protection Agency (EPA) Cybersecurity Assessment Resources. The most frequently used NIST Cybersecurity Framework with Risk IT assessment in water/wastewater technology
Failure Mode and Effects Analysis^73,88,89^	Proactive risk identification, improved reliability and safety, compliance with regulations and policies, cost reduction associated with downtime, repairs, and warranty claims	Dependence on team experience with risk assessment, intensive HR resources, potential for overlooked failures, subjectivity	Combined with Hazard and Operability Study (HAZOP), Fault Tree Analysis (FTA), Event Tree Analysis (ETA), ISO Quality Management Systems (ISO9001), and Six Sigma (SS), Project Management Body of Knowledge (PMBOK), these frameworks provide a comprehensive approach to identifying, analyzing, and mitigating risks in water and wastewater systems. In water/wastewater technology, the framework is combined with HAZOP, NIST Cybersecurity model, ISO31000, EPA Cybersecurity Assessment Resources, Environmental Risk Assessment Models
FTA^50,81,82,90–102^	Systematic approach, visual representation, identification of root causes, quantitative analysis, integration with other methods	Detailed data set requirements with accurate data on system compounds and failure rates which are challenging to obtain	Failure Mode and effects Analysis, Monte Carlo Simulation, Reliability Block Diagrams (RBD) helps in dynamic assessment of system reliability by breaking them down into simpler subsystems and components, Markov Analysis that includes dynamic assessment of system reliability by considering the probabilities of transitioning between different states over the time.
ETA^50,82,99,101–103^	Clear visualization, structured approach, quick learning curve, identification of improvement	Single initiating event, extensive for long paths that are very complex for longer event sequences, static nature, no standard graphical representation	FTA, MCS, Fuzzy Logic, Hazard and Operability Study, Risk Matrices and Checklists
Quantitative Microbial Risk Assessment Framework^86,104–112^	Preventive measures, systematic framework, data-driven decisions, versatility, risk communication	Uncertainty and variability, complexity, limited dose–response data, implementation challenges, data requirements	Computational Fluid Dynamics (CFD), LCA, Environmental Impact Assessment (EIA), WSP, HAZOP
Macrolevel Risk Models			
Task Force on Climate-related Financial Disclosures (TFCFD) - Aligned Climate Risk Disclosure Framework^113–116^	Structured and consistent reporting; investor relevance, alignment with global standards; regulatory integration; transparency and accountability	Financial materiality focus; scenario analysis challenges, complexity and resource intensity; the framework may require updates, creating a moving target for compliance	Combined with scenario analysis, integrated risk management, and sometimes Multi-Criteria Decision Analysis (MCDA)
Strengths, Weaknesses, Opportunities, and Threats (SWOT) analysis^74,117,118^	Simplicity and ease of use, comprehensive overview, promotion of strategic thinking, versatility, enhanced team communication	Oversimplification, subjectivity, lack of prioritization without balanced focus, static nature without counting all dynamic changes in the environment	Combined with Political, Economic, Social, Technological, Legal, and Environmental (PESTLE) analysis, Risk management strategies, scenario planning, Porter’s Five Forces, Environmental Risk Assessment Framework
Political, Economic, Social, Technological (PEST) Analysis^75,119,120^	Comprehensive understanding, informed decision-making, proactive risk management, alignment with regulatory requirements, identification of multifold opportunities	Oversimplification, time and HR resource intense, static nature that does not account for rapid changes in the external environment, data accuracy, subjectivity, influenced by perspectives and biases of the individuals conducting the analysis	It was combined with ISO31000, SWOT, NIST Cybersecurity Framework, Environmental Risk Assessment
Scenario Analysis Framework^76,121,122^	Alignment to stakeholders’ objectives, fostering better communication, flexibility and adaptability, management of risks toward their mitigation, enhanced decision-making by considering multiple potential futures to make the decision process more informed and strategic	Static nature without considering the dynamic changes in the technology and IT sector, HR intensive, potential to overlook risks, a large data set is required to develop accurate and meaningful scenarios, managing and analyzing multiple scenarios can be complex, especially when large data sets and numerous variables are included	Combined with sensitivity analysis, Monte Carlo Simulation, SWOT, PESTLE, FMEA, ISO31000
Cost Benefit Analysis Framework^77,123,124^	Quantitative evaluation, informed decision-making, resource allocation, transparency, sustainability and opportunity to plan in a long-term	Data accuracy that depends on the data quality and reliability, intangible factors which are not easy to quantify, e.g., social benefits, environmental impact, employee satisfaction, complexity and time- and HR-consuming requiring significant expertise and resources, static nature without considering changes in technology and supply chain	Combined with environmental assessment, life cycle assessment, risk-based evaluation models, uncertainty analysis
Life Cycle Analysis^79,125–134^	Comprehensive Environmental Impact Assessment, Resource Efficiency, Informed Decision-Making in the team, policies and standards compliance, identification of improved opportunities which can be reassessed in the following LCA	Data intensity with respect to search for accurate data, complexity requiring knowledge and understanding LCA approach, static nature, subjectivity due to the possibility to change the assumptions	Quantitative risk assessment, environmental impact assessment, geographic information system, cost-benefit analysis, multicriteria decision analysis
Life Cycle Cost Assessment^18,19,80,135–137^	Comprehensive cost evaluation, sustainability, informed decision-making, HR resource optimization, enhanced budgeting and planning, opportunity to combine with life cycle analysis to calculate the emissions, and with risk assessment through multiple scenarios	It requires a very specific data set that is not available conventionally requiring more communication with stakeholders, uncertainty in prediction, initial cost focus, subjectivity in assumptions, complexity requiring education and knowledge how to perform the analysis	It can be combined with environmental impact assessment, life cycle assessment, cost-benefit analysis, geographic information system, multicriteria decision analysis. It is combined in parallel with the life cycle analysis that can include either life cycle risk assessment or multiple-criteria assessment
Markov Analysis^68,83,138–140^	Simplicity, Out-of-Sample Forecasting, Versatility, Efficiency	Limited explanatory power, assumption of memory lessness, statis nature. Not suitable for all systems (especially when a system is too complex)	Monto Carlo Simulation, Fault Tree Analysis, Bayesian Network, RBD, Dynamic Risk Assessment
Integrated Risk Analysis^84,85,141–143^	Adaptability to many various scenarios, enhanced risk communication, improved decision making, holistic view	Resource-intensive leading to complexity, data requirement, uncertainty management, integration challenges	Quantitative Microbial Assessment, Hazard and Operability Study, Life Cycle Assessment, Bayesian Network, Multicriteria Decision Analysis
Both Micro- and Macrolevel Risk Models			
Monte Carlo Simulation^81,90,144–146^	Handling uncertainty, flexibility, detailed risk analysis, improved decision-making, visualization of results	Data quality, computational intensity, complexity in interpretation, assumption of sensitivity, resource intensive	Gray Analytic Hierarchy Process (G-AHP), Criteria Importance Through Intercriteria Correlation (CRITIC) method, Artificial Intelligence (AI) and machine learning (ML), Finite Mixture Models, Comprehensive Water Quality Index
Multicriteria Decision Analysis^78,122,147^	Comprehensive evaluation of multiple criteria simultaneously providing a holistic view of the decision problem, structured decision-making, stakeholder involvement, flexibility enhanced understanding	Subjectivity, data requirements challenging to obtain extensive and accurate results, analysis can be complex when dealing with numerous criteria and alternatives, simplification of real-world situation, HR resource intensive	It can be combined with LCA, geographic information systems (GIS) to perform the spatial analysis of risks in urban waterlogging, EIA, risk-based evaluation models to assess the cost-effectiveness and suitability of different water and wastewater technologies

Failure Mode and Effects Analysis (FMEA), described by ref 73, provides a systematic approach for identifying potential failure modes within a system and assessing their effects on system performance to prioritize risks based on their severity, occurrence, and detectability, enabling proactive mitigation strategies. Similarly, SWOT (Strengths, Weaknesses, Opportunities and Threats) analysis, utilized by ref 74, helps organizations understand internal and external factors that can impact their operations and strategic planning. In the literature, SWOT analysis is very often followed by the PEST (Political, Economic, Social, and Technological) framework, employed by ref 75, which aids in understanding the broader context of water systems operations and the identification of potential external risks. The Scenario Analysis Framework, discussed by ref 76, explores different future scenarios and their potential impacts on water and wastewater systems, supporting organizations in the preparation for various contingencies and development of robust risk management strategies.

The Cost-Benefit Analysis Framework (CBAF), emphasized by ref 77, evaluates the economic feasibility of risk mitigation measures to compare the costs and benefits of different risk management options, ensuring that resources are allocated efficiently. Multi-Criteria Decision Analysis (MCDA)^78^ evaluates multiple risk factors and decision criteria simultaneously, facilitating the prioritization of risk mitigation actions based on a comprehensive assessment.

LCA, highlighted by ref 79, assesses the environmental impacts of water and wastewater technologies throughout their life cycle identifying areas for improvement and reducing the overall environmental footprint. Life Cycle Cost Assessment (LCCA) is very often an addition to the LCA assessment evaluating the total cost of ownership of water and wastewater technologies that includes initial costs, operation and maintenance expenses, and end-of-life costs.^80^

The Fault Tree Analysis (FTA) framework, described by ref 81, being a top-down approach for identifying potential causes of system failures, has been extensively used to visualize the pathways leading to failures and developing prevention strategies. However, there is a significant opportunity to enhance risk assessment in the water and wastewater sector by integrating advanced technologies with a focus on real-time data integration, predictive analytics, and dynamic modeling to address the evolving challenges in this critical sector. In water and wastewater risk management, Event Tree Analysis (ETA) is less popular than FTA because it pinpoints specific failures and system breakdowns. This makes FTA essential for detailed hazard assessments and accident investigations, especially since risk assessment in the water and wastewater sector has long been associated with these issues.^82^ ETA risk assessment focuses on understanding the potential outcomes and effectiveness of the safety measures.

Markov analysis, remaining a popular risk assessment framework since the beginning of the 21st century, models the probabilistic behavior of systems over time helping to predict the future states of water and wastewater systems and assess the long-term risks.^83^ On the contrary, the integrated risk frameworks, emphasized by refs 84 and 85 , integrate various quantitative and qualitative risk assessment methods to provide a comprehensive evaluation of risks that has not been developed significantly since 2006. The framework is designed to address the complexity of engineering systems, manage resource intensity, and adhere to standardized regulations. However, due to the inherent complexity of the water and wastewater sector, it often fails to fully address these issues.^36^

The Quantitative Microbial Risk Assessment (QMRA) framework assesses the risks posed by microbial contaminants in water systems evaluating the likelihood of exposing to pathogens and potential health impacts supporting the development of safety standards.^86^ This framework has been widely adopted by industrial sites because it provides accurate data analysis on pathogen concentrations and exposure levels. The resulting insights into human health and welfare are essential for achieving governmental standards and policies. Monte Carlo Simulation (MCS) is very often combined with other risk assessment frameworks, such as ETA and FTA, to assess uncertainty and variability of the overall model by exploring different scenarios and evaluating the probability of various outcomes.^81^ The combined use of Quantitative Microbial Risk Assessment (QMRA) and Monte Carlo Simulation (MCS) is essential for comprehensive risk management in the water and wastewater sectors, providing a more holistic understanding of risks and uncertainties. However, this combination still faces limitations, due to the ability to address a limited range of risk categories, including environmental, technological, and operational risks.^87^

A diverse range of risk assessment frameworks is available for water and wastewater systems, highlighting their complexity and multifaceted nature to manage these critical infrastructures (Table 2). Each framework offers unique advantages and addresses specific aspects of risk, from technological and IT-related threats to environmental impacts and microbial contamination. However, only the combination of these frameworks can achieve a more comprehensive and robust risk assessment in water and wastewater management, ensuring safety, reliability, and sustainability due to the inclusion of diverse risk categories, as discussed in Section 3.1. Continuous development and adaptation of these frameworks will address emerging challenges and leverage advancements in technology and data analytics, ultimately contributing to the protection of public health and the environment, while also supporting balanced regional economies, optimal manufacturing conditions, and the rapid development of sociocultural trends in EU countries.

As the complexity of water systems increases, these frameworks will need to evolve to address new challenges such as climate change, aging infrastructure, and new contaminants. In parallel, the global shift toward circular water systems, which include water reuse, resource recovery, and closed-loop industrial processes, adds new layers of complexity that risk frameworks must be equipped to handle.

The shift from linear to circular water systems represents a fundamental transformation in how water is managed, valued, and governed. Traditional risk frameworks, which often focus on isolated technical or financial risks, are insufficient for capturing the complexity and interdependencies inherent in circular water economies. Instead, a new generation of risk assessment models must be capable of evaluating circularity across technological, economic, sociocultural, and regulatory dimensions.

The previous work^148^ emphasized that circular water technologies (CWTs) are not only about technical innovation but also about systemic change, underscoring the importance of combined micro- and macrolevel risk assessment. These technologies aim to recover water, energy, and materials from wastewater, thereby creating value chains that extend beyond the water sector into agriculture, energy, and industry. However, the uptake of such technologies is often hindered by fragmented governance, unclear regulatory frameworks, and limited public acceptance. Therefore, risk frameworks must assess not only the technical feasibility of CWTs but also their integration into broader socioeconomic and policy contexts.

Another work^149^ proposed the Multi-Sectoral Water Circularity Assessment (MSWCA) framework, which models the interactions between human-managed and nature-managed systems. This combined micro- and macrolevel risk framework integrates material flow analysis (MFA), LCA, and economic valuation to assess circularity performance. It also incorporates feedback loops and interdependencies between sectors, enabling a holistic understanding of how changes in one part of the system may affect others. For instance, nutrient recovery from wastewater must be evaluated not only for its technical efficiency but also for its market viability, environmental impact, and social acceptance.

The use of causal digital loop diagrams enables visualization of how these risks affect performance metrics such as efficiency and cost. Their taxonomy of 40 Circular Economy (CE)-related risks, combined with participatory modeling, supports proactive decision-making and enhances resilience by aligning risk management with circularity goals.^150^

All of these studies highlight the importance of circularity performance indicators (CPIs) and establishment of circularity databases, which serve as action-oriented metrics to guide decision-making. These indicators span resource flows, societal values, and system properties, aligning with the principles of a circular economy: regenerating natural capital, keeping resources in use, and designing out waste. Risk frameworks must therefore be equipped to measure these indicators and interpret them in the context of long-term sustainability and resilience.

The integration of real-time data analytics, predictive modeling, and dynamic simulations is crucial in this evolution (Table 1). One such advancement is the incorporation of the Task Force on TCFD-aligned Climate Risk Disclosure Framework, which emphasizes the need for transparent, consistent, and comparable climate-related risk reporting.^115,116^ This framework encourages organizations, including those in the water sector, to assess and disclose climate-related risks across four key areas: governance, strategy, risk management, and metrics and targets. By aligning risk assessment frameworks with TCFD principles, water utilities can better understand and communicate the financial implications of climate risks such as extreme weather events or long-term shifts in water availability.

AI can enhance the predictive capabilities of frameworks such as FMEA and FTA by analyzing vast amounts of data to identify patterns and predict potential failures before they occur, reducing the risk of system failures and improving the overall reliability of water systems. By creating digital twins of water and wastewater systems, operators can simulate different scenarios and assess the impact of various risk factors in a controlled environment. This technology can be particularly useful in scenario analysis frameworks, allowing for more accurate and comprehensive risk evaluations. The use of IoT devices will further enhance the capabilities of risk assessment frameworks. IoT sensors can provide real-time data on water quality, flow rates, and system pressures, which can be integrated into risk models to provide up-to-date risk assessments. This real-time data integration will enable more responsive and dynamic risk management strategies, ensuring that water systems can quickly adapt to changing conditions. Moreover, the combination of AI and IoT can improve the effectiveness of frameworks, such as Quantitative Microbial Risk Assessment, by providing more accurate and timely data on microbial contaminants. AI algorithms can analyze these data to predict the likelihood of contamination events and suggest appropriate mitigation measures, thereby enhancing the safety of water supplies and further optimizing the uncertainty using the Monto Carlo Simulation.

There are several examples of companies and municipalities implementing these integrated approaches. For example, Epic Cleantec provides distributed wastewater treatment with onsite water reuse for commercial or large residential buildings, recycling up to 95% of a building’s wastewater. The benefit of the combined risk frameworks is not only enhancement of sustainability but also reduction of dependence on freshwater sources. However, interdependencies with other critical infrastructures, cybersecurity threats, public perception, community engagement, and sociocultural factors have not been integrated into this combined framework for wastewater treatment plants and are also rarely addressed in other risk assessment frameworks. While a single, standardized risk framework may not yet be established, the trend is toward integrating multiple advanced technologies and methodologies to create more comprehensive and effective water and wastewater management systems. The continuous development and adaptation of these frameworks, combined with advancements in digital technologies and AI, will play a crucial role in addressing the complex challenges faced by the water and wastewater sectors in the future.

The microlevel risk assessment model implemented at the Irish facility represents a proactive and comprehensive approach to managing environmental and operational risks in wastewater treatment. It integrates environmental monitoring, hazard identification, real-time data analysis, and scenario-based planning to ensure compliance, safety, and sustainability in a sensitive ecological context.

Looking ahead, several recommendations can enhance the model’s effectiveness. First, integrating regulatory and policy risks more dynamically into the core structure would allow for a more responsive compliance management. Second, leveraging predictive analytics and machine learning on SCADA and laboratory data could help anticipate failures or compliance breaches before they occur. Third, expanding training programs based on real-world scenarios from the risk model would improve staff preparedness and operational resilience. Additionally, involving local environmental agencies and community stakeholders in periodic reviews could strengthen the transparency and alignment with broader sustainability goals. Finally, implementing a digital twin of the WWTP could enable virtual testing of risk scenarios and mitigation strategies, further enhancing the robustness of the risk management framework.

### 3.5 Case Studies

#### 3.5.1 Microlevel

Since 2020, a powdered nutrition manufacturing facility located in Ireland, has strengthened its operational risk management by implementing a structured microlevel risk assessment model for WWTP that was designed to handle a daily flow of 2,800 m^3^/day of wastewater with a maximum flow rate capacity of 126 m^3^/hour. The site, which produces powdered infant and maternal nutrition products, operates in a highly regulated and environmentally sensitive area near the Shannon estuary. This region is characterized by karstified bedrock and interconnected groundwater systems, necessitating robust environmental controls. Figure 3 shows the schematic diagram representing the WWTP detailing the flow and treatment stages of wastewater originating from food manufacturing.

**Figure 3:**
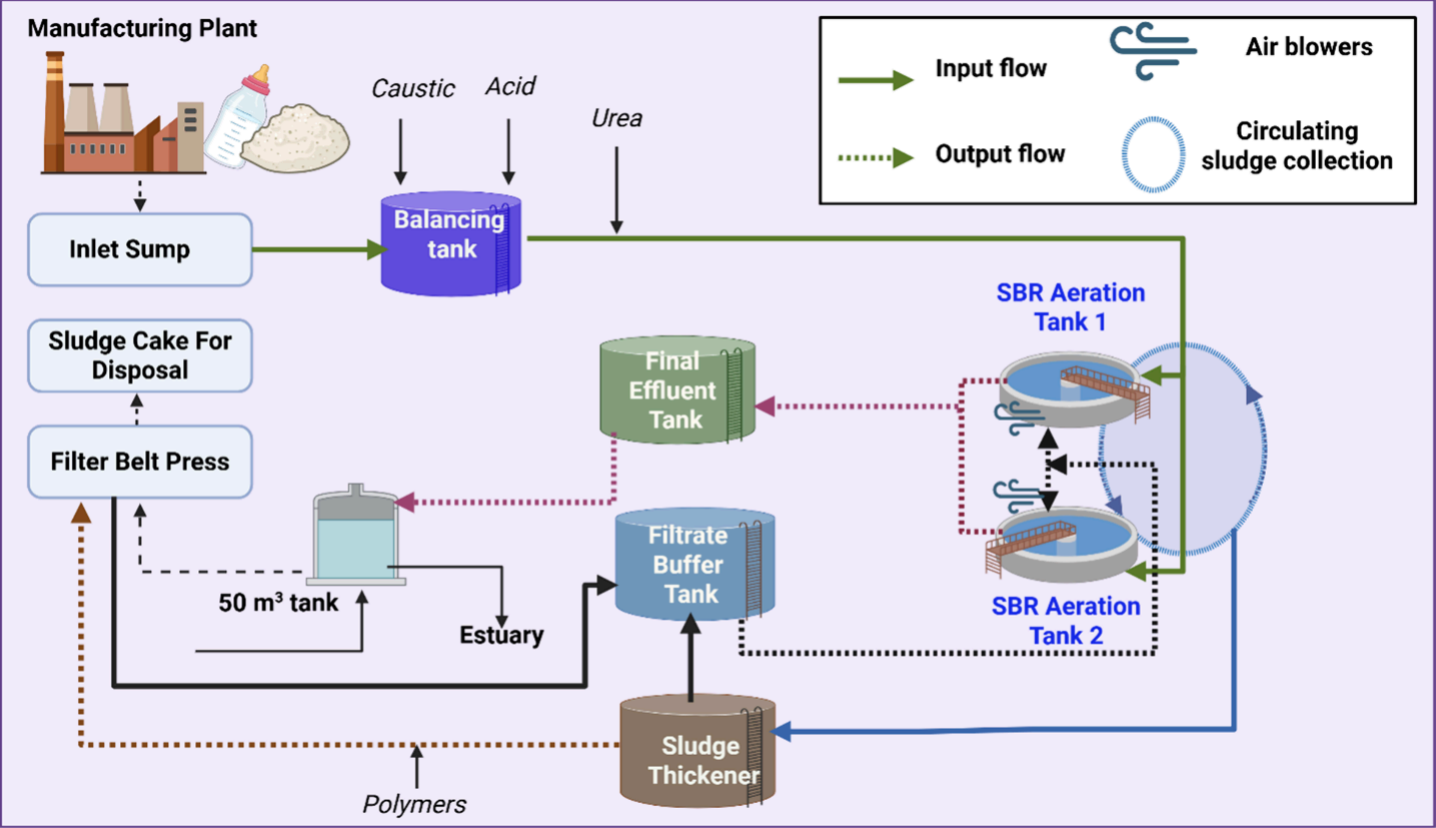
WWTP presented schematically.

The process begins at the inlet sump, where the raw wastewater is collected. From there, it is directed to a balancing tank, which serves to equalize fluctuations in flow and composition, ensuring a consistent feed to the biological treatment stage. The core of the treatment process involves two sequential batch reactors for the biological treatment of wastewater using aeration to promote the breakdown of organic matter by microorganisms. Once treated, the water flows into the final effluent tank serving as a storage before water transfer into a 50 m^3^ tank. The final discharge occurs at the estuary discharge point, where the treated effluent is released into the environment.

Sludge generated during the treatment process is managed separately. It is first thickened in a sludge thickener and then further dewatered using a filter belt press, producing a sludge cake suitable for disposal. The water separated during this process, known as a filtrate, is collected in a filtrate buffer tank. The system also includes various chemical dosing units such as caustic, acid, polymer, and urea, primarily to adjust the carbon-to-nitrogen (C/N) nutrient ratio, with secondary benefits such as enhancing buffering capacity and supporting coagulation and nutrient balance. Air blowers provide the necessary oxygen for biological processes, and a portable part of the water is treated on site.

The WWTP risk assessment model is based on a task-specific framework that identifies and evaluates operational hazards associated with wastewater treatment processes. As illustrated in Figure 4, the model incorporates key environmental factors such as temperature, pH, oxygen levels, nutrients (N, P), and toxicity, which directly influence microbial activity within the treatment system. In addition to these environmental influences, the model also considers operational hazards such as biological agents, chemical exposure, confined space entry, ergonomic strain, and vehicle-related risks. Each hazard is assessed using a structured matrix approach that evaluates both probability and severity and applies to a hierarchy of controls ranging from engineering solutions and administrative procedures to personal protective equipment (PPE) to mitigate risks effectively. The microlevel risk model for water and wastewater management is shown in Figure 5.

**Figure 4:**
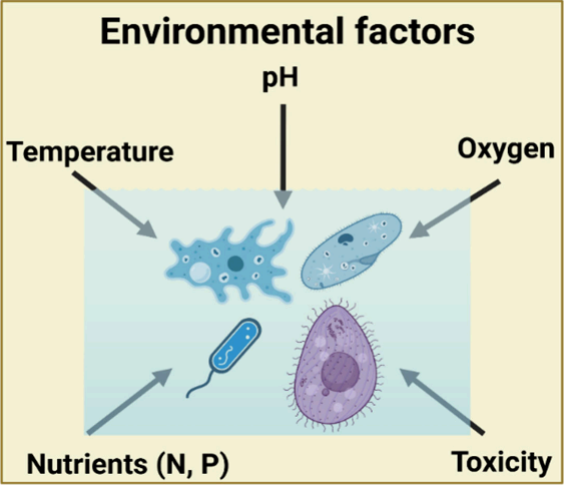
Environmental factors used in the microlevel risk model.

**Figure 5:**
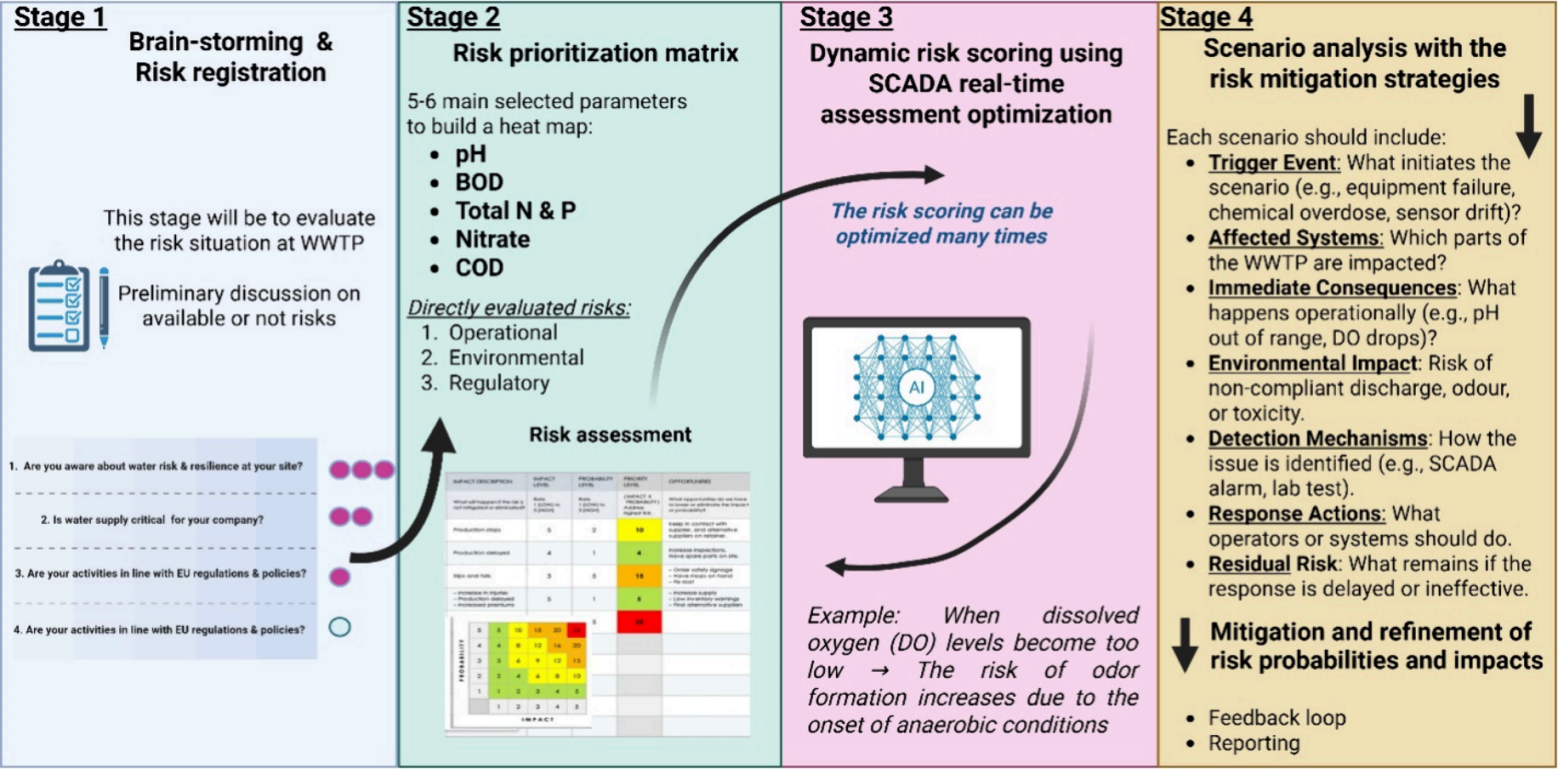
Microlevel risk model.

The four-stage risk assessment framework for a WWTP reflects a broader trend in the food manufacturing sector, where risk assessments are predominantly centered on operational, environmental, and regulatory domains (Figure 5). Beginning with brainstorming and risk registration, the model prompts organizations to evaluate their awareness of water-related risks and regulatory alignment, particularly in contexts where water supply is critical to operations. This is followed by a risk prioritization matrix that focuses on key parameters such as pH, biological oxygen demand (BOD), total P and N, Nitrates, and chemical oxygen demand (COD) metrics essential for maintaining compliance and environmental stewardship, especially in export-driven industries where traceability and hygiene are paramount.

The outcome from the risk prioritization assessment and subsequent Supervisory Control and Data Acquisition (SCADA) based real-time monitoring culminated in a structured scenario analysis and the implementation of targeted mitigation strategies. Each scenario was developed with detailed components, including trigger events (such as equipment failure or chemical overdosing), affected systems, immediate operational consequences, and potential environmental impacts like noncompliant discharge or odor release.

Detection mechanisms ranging from SCADA alarms to lab tests enabled the timely identification of issues, while predefined response actions ensured swift intervention. This process was reinforced by a continuous feedback loop and systematic reporting, which not only minimized risk but also established a resilient framework for long-term control and monitoring of the WWTP facilities.

While the microlevel model does not explicitly account for regulatory and policy risks, these considerations are incorporated during the initial brainstorming stage by embedding compliance criteria into the risk prioritization matrix. Additionally, during the risk prioritization process, measured parameters from the WWTP facilities are evaluated against thresholds set by the EU and national regulations. If these values exceed regulatory limits, the system triggers a reassessment loop, prompting a re-evaluation of potential risk sources across the entire WWTP assessment framework.

The microlevel risk assessment model implemented at this Irish facility represents a proactive and comprehensive approach to managing environmental and operational risks in wastewater treatment. It integrates environmental monitoring, hazard identification, real-time data analysis, and scenario-based planning to ensure compliance, safety, and sustainability in a sensitive ecological context. Integrating regulatory and policy risks more dynamically into the core structure would allow for a more responsive compliance management. Leveraging predictive analytics and machine learning on SCADA and laboratory data could help to anticipate failures or compliance breaches before they occur. The expansion of existing training programs based on real-world scenarios from the risk model can improve staff preparedness and operational resilience. The implementation of a digital twin for risk monitoring at the WWTP could enable virtual testing of risk scenarios and mitigation strategies, further enhancing the robustness and continuity of the risk established management framework.

#### 3.5.2 Macrolevel

Since 2023, the Portuguese manufacturing company has enhanced its risk management process by implementing a new Enterprise Risk Management (ERM) system, adhering to Committee of Sponsoring Organizations (COSO) and ISO31000 guidelines.^151,152^ The macrolevel risk assessment model is based on the case study of the EU manufacturer producing uncoated printing, writing paper, and bleached eucalyptus pulp. The company is committed to sustainable practices and innovative solutions, integrating environmental stewardship and ensuring responsible management of forest resources. All paper and pulp sites operate 50000 to 60000 m^3^ d^–1^ capacity equipped WWTPs. The mills have their own water collection and effluent treatment systems. Water collection is done in surface or underground environments. Effluent treatment systems consist of primary and secondary treatments, and one of the WWTPs is equipped with an ultrafiltration system. Effluents are discharged into the sea, through underwater outlets, or into an estuary.

The Risk Management Committee updated a new Risk Management Governance Model that focuses on continuous risk monitoring, empowering the risk management process, leveraging specific expertise, and coordinating various risk management areas. Five subcommittees (Commercial, Production, Skills, Resilience, and Reputational) were established to integrate and coordinate risk relations, calibrate risk assessments, monitor and promote risk mitigation, provide expertise, implement mitigation measures, and communicate risk issues. This model includes business units managing daily risk activities, several divisions that support risk management implementation and monitoring, and audit units (The Internal Audit Office, Audit Board, and Risk Management Board) which supervise compliance with policies and standards.

The risk management process follows internationally accepted risk management best practices, models and frameworks.^153,154^ Figure 6 shows a process that consists of a set of seven interrelated phases, encompassing an iterative process of continuous improvement, embodied by a communication and consultation process and a monitoring and review process.

**Figure 6:**
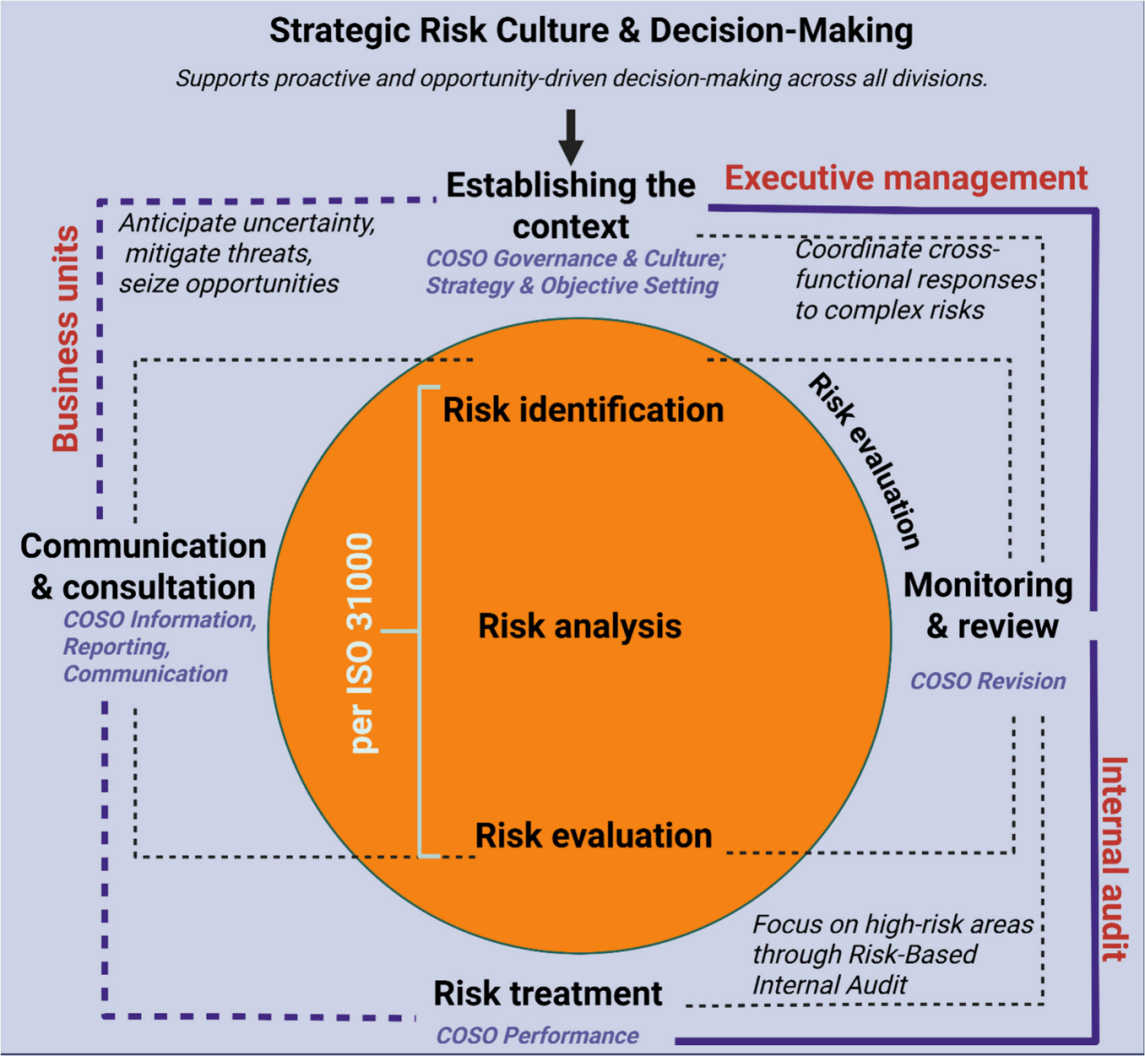
Flow of the risk management process at the corporate level.

Figure 7 shows the prioritization matrix that provides a clear visual representation of risks, helping organizations make informed decisions by determining which risks require immediate attention and resources and which can be monitored with less urgency. The risk prioritization matrix was established through surveys and interviews, historical data analysis, and external sources obtained at various industrial sites across Europe. Systematically collected and analyzed data was used to establish a prioritization matrix by addressing risks at both corporate and medium management levels. The identified risks that are both highly probable and have severe impacts require immediate attention and robust mitigation strategies. Risks with moderate probability and impact are less critical but still require monitoring and management including supply chain disruptions and workforce shortages which affect the continuity of operations and overall process efficiency. Mitigation strategies for moderate risks include diversifying suppliers, maintaining adequate inventory levels, and investing in workforce development programs.

**Figure 7:**
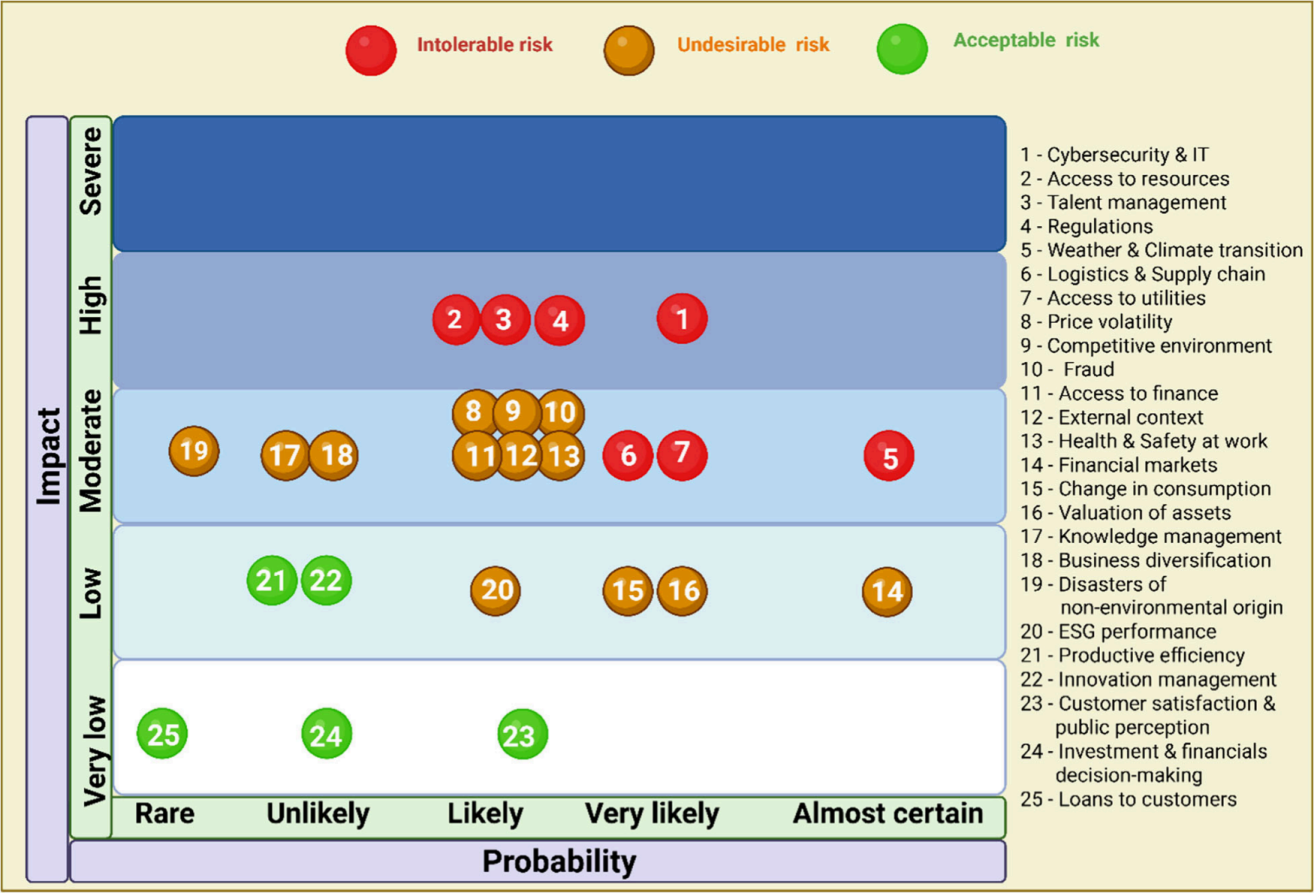
Prioritization risk matrix.

Risks that are unlikely to occur but would have severe consequences need contingency plans to ensure preparedness. Natural disasters and cybersecurity threats occur rarely, but their impact can be devastating, necessitating comprehensive disaster response and recovery plans as well as robust cybersecurity measures. It is important to highlight the risk category *Access to Utilities (7)* which includes the risk related to water access, as such disruptions, though infrequent, can critically impair operations. Similarly, the *Regulations* category encompasses the risk of noncompliance with environmental legislation, which, while potentially rare, can lead to severe legal and reputational consequences. These risks require proactive strategies to ensure compliance and resilience. On the other hand, risks that are both unlikely and have minimal impact must be monitored to ensure they do not escalate but generally do not require significant resources for mitigation.

The prioritization matrix has been used to assess the holistic risks at the company, but several attempts were made to use this comprehensive approach for managing specifically water-related risks that correspond to the TCFD risk analysis framework.^114^ This approach does not delve into microlevel risk assessments that focus on individual operational processes or site-specific vulnerabilities. Instead, it addresses the broader implications of climate change on water availability and quality, particularly in the hydrographic regions where the company operates. As such, the focus remains on strategic, high-level risk evaluation rather than on detailed, process-based risk management. Several water-related risks have been identified and evaluated in detail at a systemic level, including physical risks such as droughts, water shortages, water stress, floods, saltwater intrusion, and rising sea levels as well as transition risks related to regulatory and technological changes. These risks affect both the supply chain and the company’s own use of water resources. The company corporate strategy was reported in the 2030 Roadmap by outlining specific goals, such as reducing water use by 33% from the 2019 baseline and decreasing the organic load in effluents by 10% per ton of output from the 2022 baseline. This strategy was thoroughly incorporated into the risk assessment conducted as part of the current case study, illustrating how Enterprise Risk Management (ERM) frameworks are being extended to incorporate water-related risks at both the corporate and site levels. However, a key limitation remains in the current approach that does not fully integrate these risks into operational- or process-level decision-making.

Additionally, the company is committed to monitoring the impact of bioresource conversion processes on water management and exploring ways to reduce water consumption at its nurseries. At the core of the 2030 Roadmap, community engagement is a key aspect of the corporate water risk management strategy, ensuring that the company’s operations do not pose additional threats to the environment and surrounding communities. Furthermore, the company has included in its framework the financial impact of climate change on water-related risk management, aiming to balance operational efficiency, sustainability, and economic viability in decision-making.

Participation by external stakeholders is a key feature of the risk assessment, exemplified by their involvement in bodies such as the Environmental Council comprising independent academics with expertise in environmental matters and the Ethics and Integrity Committee, which includes respected external figures tasked with overseeing adherence to the Code of Ethics and Conduct. Figure 8 highlights how robust compliance frameworks ranging from ethics and anticorruption to human rights and third-party integrity are essential for managing macrolevel risks. External stakeholders, such as suppliers, regulators, and international partners, play a vital role in this process. Their actions, values, and compliance with shared standards directly influence the effectiveness of risk mitigation strategies and contribute to broader goals like United Nations (UN) Sustainable Development Goals (SDG) 16: Peace, Justice, and Strong Institutions. Together, these mechanisms ensure that risk modeling strategy is informed by diverse perspectives, science-based targets, and a commitment to long-term sustainable value creation.

**Figure 8:**
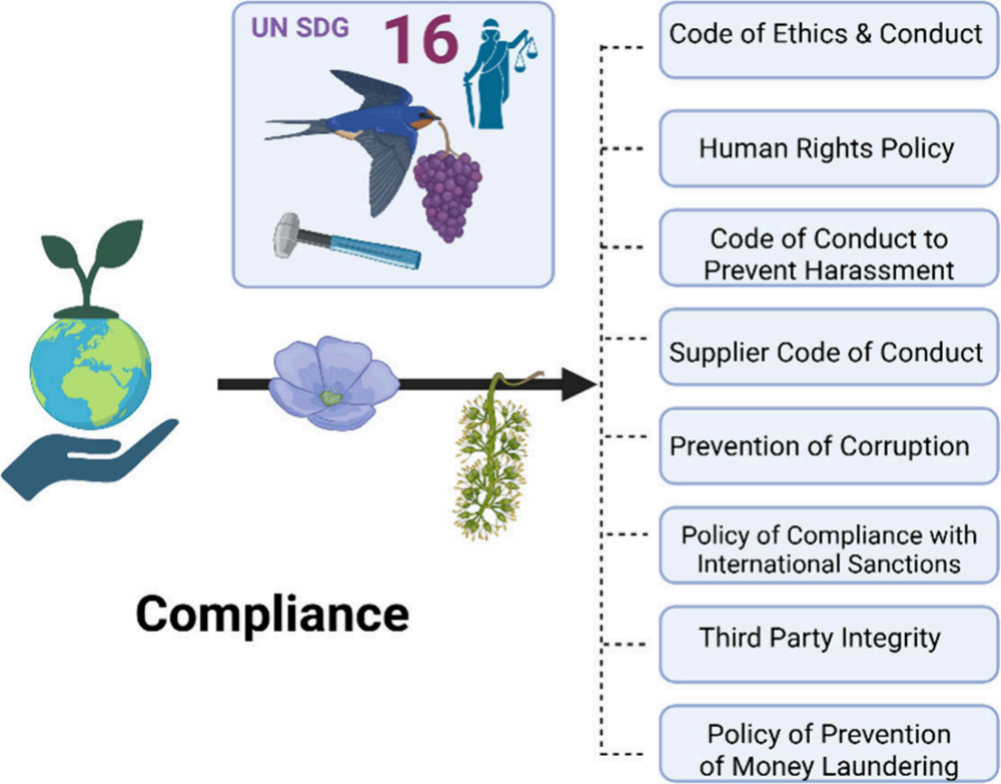
Compliance system and fundamental instruments following UN SDG16.

The next step will be the integration of a bottom-up approach to prepare microlevel risk assessments at various water basins and manufacturing sites and relate these to the established corporate or macrolevel model.

### 3.6 Future Perspective

The increasing complexity of industrial water and wastewater systems, coupled with evolving environmental regulations and climate-related uncertainties, necessitates a shift toward more integrated and technologically advanced risk assessment frameworks. The future most effective risk management strategies will rely on the seamless integration of micro- and macrolevel models into a unified, adaptive framework. This combined approach enables organizations to align day-to-day risk assessments with long-term strategic planning, ensuring that granular insights are interpreted within a broader economic and systemic context. The integration of artificial intelligence, real-time monitoring, and blockchain technologies can enhance the capacity of both micro- and macrolevel models to manage risks adaptively across the dynamical water cycle.^155^ AI plays a pivotal role at this level by enabling predictive maintenance and early detection of anomalies, while machine learning algorithms can analyze sensor data to forecast equipment failures or chemical imbalances followed by the simulation of risk scenarios and test mitigation strategies in a controlled environment, enhancing preparedness and decision-making.^156^

Future risk frameworks will be built on shared data platforms and AI-driven decision support systems that allow microlevel insights to inform macrolevel forecasts and vice versa. For instance, a sudden increase in contaminant levels detected at a treatment plant could trigger a regional risk reassessment, while long-term drought projections could lead to adjustments in the whole organizational strategy by impacting policy-related and reputational risk thresholds. This bidirectional flow of information will enable more responsive, resilient, and efficient systems.

Integrating real-time monitoring throughout the entire water cycle offers a powerful enhancement to existing risk models by enabling continuous data collection and processing.^157^ IoT-enabled sensors can be strategically deployed at key points from source water to effluent discharge providing a constant stream of data to inform adaptive risk models.^158^ When combined with artificial intelligence, the data enables dynamic, real-time risk scoring and the automation of response protocols, reducing the lag between threat detection and mitigation.

Pilot and large-scale projects will play a key role in testing such dynamic risk models in real-world conditions, allowing for iterative refinement.^137^ Enabled by advances in cloud computing, edge analytics, and interoperable data standards, this integrated and adaptive approach will be essential for managing risk in an increasingly uncertain and interconnected world.^159^ This approach not only improves the robustness of risk models but also builds confidence among stakeholders who rely on these tools for critical decision-making.

This article presented a microlevel risk case study by focusing on the operational and environmental hazards within specific facilities, such as WWTPs. These models are typically task-specific and emphasize real-time monitoring of parameters like pH, alkalinity, iron, chlorine, COD, solid loads, and BOD. This study presented the current way how the microlevel models are structured around SCADA systems, laboratory data, and scenario-based planning to ensure operational resilience. The literature shows a range of various parameters which can be measured by industrial sites to evaluate the WWTP status.^160^ Some measurements have been done manually, but others were performed semiautomatically. Manual assessments, particularly for determining mineral and halogen concentrations in wastewater, commonly employ techniques such as Inductively Coupled Plasma Mass Spectrometry (ICP-MS) and Ion Chromatography (IC), which are standard practices in water quality control.^161^ Although efforts are being made to develop inline or automated versions of these techniques, they are not yet widely adopted due to their complexity, maintenance demands, and high cost.^162^ Furthermore, sensor fouling, calibration drift, and the need for periodic validation still necessitate human oversight. Variations in national regulatory frameworks across EU member states often require independent verification or certified laboratory testing for specific critical parameters, thereby constraining the scope for automation.^163^

In contrast, the macrolevel risk model adopts a broader, enterprise-wide view, encompassing strategic, regulatory, reputational, and financial risks. The case study of a European pulp and paper manufacturer illustrates how macrolevel frameworks, such as those aligned with ISO 31000 and COSO ERM, can be used to prioritize risks across multiple sites and integrate sustainability goals into corporate strategy. These models are particularly effective in addressing systemic risks such as climate-induced water scarcity, regulatory shifts, and supply chain disruptions. AI supports macrolevel assessments by aggregating and analyzing data from diverse sources of internal audits, environmental monitoring, and stakeholder feedback to identify emerging trends and inform long-term planning. Additionally, macrolevel models benefit from stakeholder engagement and governance structures that ensure accountability and alignment with broader sustainability frameworks, such as the SDGs.

Equally transformative for water governance is the application of blockchain technology to establish a verifiable chain of evidence in water risk management. Blockchain can record every transaction, measurement, and corrective action in an immutable ledger, enhancing transparency and traceability. This is particularly valuable in regulated industries, where documentation of compliance and environmental stewardship is critical. Blockchain also facilitates secure data sharing among utilities, regulators, and communities, fostering trust and collaboration in water governance.

The successful integration of AI and block-chain technology depends on solutions to overcome challenges such as data standardization, cybersecurity, and interoperability with legacy systems. Future research should focus on developing modular, interoperable platforms that combine these technologies into cohesive frameworks tailored to the specific needs of industrial sectors. Another challenge with AI and block-chain technology is public skepticism and concerns about privacy, surveillance, and job displacement. Ensuring transparency, ethical governance, and inclusive education initiatives will be crucial for fostering public trust and participation.

In addition, the uneven pace of technological adoption among European countries and industries may create economic imbalances in competitiveness and innovation capacity. Small and medium-sized enterprises (SMEs), which form the backbone of many European economies, may struggle with the high costs and technical complexity of implementing these technologies. Policymakers must therefore prioritize funding mechanisms, cross-border collaboration, and regulatory harmonization to support equitable growth and innovation across the continent.

While future water and wastewater risk models are increasingly defined by technological integration and adaptive frameworks, critical dimensions such as social engagement and economic considerations remain underexplored. Social engagement is recommended to become a core component of risk governance, ensuring that public trust, transparency, and participatory monitoring are embedded in system design requiring more surveys and publicly available documents and consulting of communities. Economic considerations, such as cost-benefit analyses and return on investment for AI and blockchain adoption, are essential for justifying large-scale implementation for SMEs including the challenge of how to estimate the true cost of the water which is not just in terms of direct operational expenses but also factoring in environmental externalities, regulatory compliance, long-term resource scarcity, and reputational risks. Traditional accounting methods often overlook these hidden costs, leading to underinvestment in advanced risk management technologies. A more holistic economic framework is needed that incorporates lifecycle costing, ecosystem service valuation, and scenario-based financial modeling to capture the full spectrum of risks and benefits. This would enable SMEs to make more informed decisions about technology adoption while also supporting policymakers in designing incentives and funding mechanisms that reflect the real value of sustainable water management. Moreover, cross-sectoral integration is vital, as water systems are deeply interconnected with agriculture, urban infrastructure, and energy networks, necessitating a holistic approach to risk that spans multiple domains. Risk models that can be validated across industrial sites by using minimal operational parameters will greatly enhance their reproducibility. Moreover, the high pressure from the EU Committee, with respect to climate adaptation, will reinforce the predictive modeling by inclusion of nature-based solutions and resilience planning that address environmental, regulatory, and social vulnerabilities.

## 4 Conclusion

This article highlights the growing complexity and critical importance of industrial water and wastewater risk assessment in the face of evolving environmental, technological, and regulatory challenges. By synthesizing a wide range of frameworks, methodologies, and case studies, it underscores the necessity of integrating micro- and macrolevel risk models to ensure both operational resilience and strategic foresight. The incorporation of advanced technologies such as AI, IoT, and blockchain, alongside stakeholder engagement and regulatory alignment, offers a path toward more adaptive, transparent, and sustainable water management systems. Future efforts should focus on harmonizing these tools within a unified, dynamic framework that supports continuous improvement, cross-sectoral collaboration, and equitable access to innovation across industries and regions.
